# GeneMarkeR: A Database and User Interface for scRNA-seq Marker Genes

**DOI:** 10.3389/fgene.2021.763431

**Published:** 2021-10-26

**Authors:** Brianna M. Paisley, Yunlong Liu

**Affiliations:** ^1^ Department of BioHealth Informatics, Indiana University-Purdue University Indianapolis, Indianapolis, IN, United States; ^2^ Toxicology, Eli Lilly and Company, Indianapolis, IN, United States; ^3^ Department of Medical and Molecular Genetics, Indiana University School of Medicine, Indianapolis, IN, United States

**Keywords:** single-cell RNA-seq1, scRNA-seq2, marker gene3, cell type4, database5, web-interface6

## Abstract

Single-cell sequencing (scRNA-seq) has enabled researchers to study cellular heterogeneity. Accurate cell type identification is crucial for scRNA-seq analysis to be valid and robust. Marker genes, genes specific for one or a few cell types, can improve cell type classification; however, their specificity varies across species, samples, and cell subtypes. Current marker gene databases lack standardization, cell hierarchy consideration, sample diversity, and/or the flexibility for updates as new data become available. Most of these databases are derived from a single statistical analysis despite many such analyses scattered in the literature to identify marker genes from scRNA-seq data and pure cell populations. An R Shiny web tool called GeneMarkeR was developed for researchers to retrieve marker genes demonstrating cell type specificity across species, methodology and sample types based on a novel algorithm. The web tool facilitates online submission and interfaces with MySQL to ensure updatability. Furthermore, the tool incorporates reactive programming to enable researchers to retrieve standardized public data supporting the marker genes. GeneMarkeR currently hosts over 261,000 rows of standardized marker gene results from 25 studies across 21,012 unique genomic entities and 99 unique cell types mapped to hierarchical ontologies.

## Introduction

scRNA-seq enables study of disease heterogeneity, novel cell subtypes, cellular interactions, and cellular tissue composition (Mancarci et al., 2017; Skelly et al., 2018; Aran et al., 2019; Saviano et al., 2020). A major challenge in scRNA-seq analysis is to identify the cell type of individual cells. Accurate cell type identification is crucial for any scRNA-seq analysis to be valid as incorrect cell type assignment will reduce statistical robustness and may lead to incorrect biological conclusions. Therefore, accurate and comprehensive cell type assignment is necessary for reliable biological insights into scRNA-seq datasets.

Marker genes, genes more specific in expression for one or a few cell types over others, are important descriptors in the identification of scRNA-seq cell type (Franzen et al., 2019; Zhang et al., 2019). Identifying marker genes can be a tedious process, and sometimes requires manual extraction from appendices and/or images of publications. Furthermore, marker genes may be specific to sample type, species, and/or sequencing technology. For example, a gene that is specific for endothelial cells in mouse brain tissue samples may not be endothelial cell specific outside of the brain or in human samples. Therefore, it is vital to improve access to accurate, robust, and translatable scRNA-seq marker genes.

The recent publication of CellMarker (Zhang et al., 2019) has provided researchers with access to marker gene lists in mouse and human. The program provides manually extracted lists of marker genes from multiple sources for users to search. While having a consolidated source of marker gene lists is helpful, researchers must still sort through data to identify which marker genes are robust and relevant to their analyses. For example, identifying species-specific markers, markers consistent across samples, and markers able to be detected in 3′-sequencing methods, would require the users to manually identify marker genes fitting their data criteria. Therefore, the primary focus of this manuscript is to provide a resource to document the marker genes that were consistently identified across species, samples, sequencing technologies, and sources.

To identify consistent marker genes for specific cell types, we manually curated results from publications that performed large-scale statistical analyses on pure cell populations via scRNA-seq or Fluorescence-activated cell sorting (FACS) methodologies. We focused on publications using expression data from mice and/or human untreated, non-disease samples. Next, the extracted gene information was standardized to known ontologies, cellular hierarchy information was incorporated, and a marker gene score algorithm to identify marker genes consistent across sources, samples, and species was developed. Two MySQL databases were generated to store: 1) the standardized, manually curated statistical results and metadata and 2) the robust marker genes, while an R Shiny reactive user-interface is provided to access the data. The development of the publicly accessible GeneMarkeR database and user-interface is described in this manuscript.

## Materials and Methods

### Data Extraction

Data curated for the database focused on publications concentrated on performing statistical analyses to identify cell type-specific marker genes in their samples. There were 25 unique marker gene analyses from these publications that either: 1) used scRNA-seq expression data, 2) used RNA-seq or microarray expression data collected from pure cell populations, or 3) came from collaborators sharing highly validated (i.e., prototypical) marker genes. Additional publications were evaluated; however, these were filtered out as the exclusive focus was on naïve (i.e., non-treated, non-disease) mouse and human samples. The marker genes, cell types and full statistical results were manually extracted from figures, supplemental data, and text of publications, or directly from the author to ensure data integrity. In a few cases only the significant marker gene results were available from the author, not the full statistical output. Additional contextual data (i.e., sample type, species, gene expression method, statistical method, relevant statistical cutoffs) were collected from each source. For publications that used scRNA-seq data, prototypical marker genes, marker genes the authors used to annotate their cell types for each cell population, were extracted. These prototypical marker genes are generally highly validated, well-accepted genes used to annotate cell types prior to performing novel marker gene identification.

### Ontology Standardization

To enable mouse-human comparison across the same genomic entity (i.e., genes, miRs, lncRNAs), Mouse Genome Informatics (MGI) and Entrez mouse-human ortholog information were used to map genomic entity information. Genomic entities for mouse (assembly GRCm39) and human (assembly GRCh38.p13) were standardized using gene symbols and unique identifiers from both Entrez and Ensembl. A unique key (GeneID) was generated to identify each unique mouse-human ortholog pair, or when no ortholog is described, to denote the mouse or human-specific genomic entity. A total of 21,012 unique genomic entities were included in the analysis. Genomic entities are referred to as genes in the Figures and Tables for readability as genes comprise most of the genomic entities.

**FIGURE 1:**
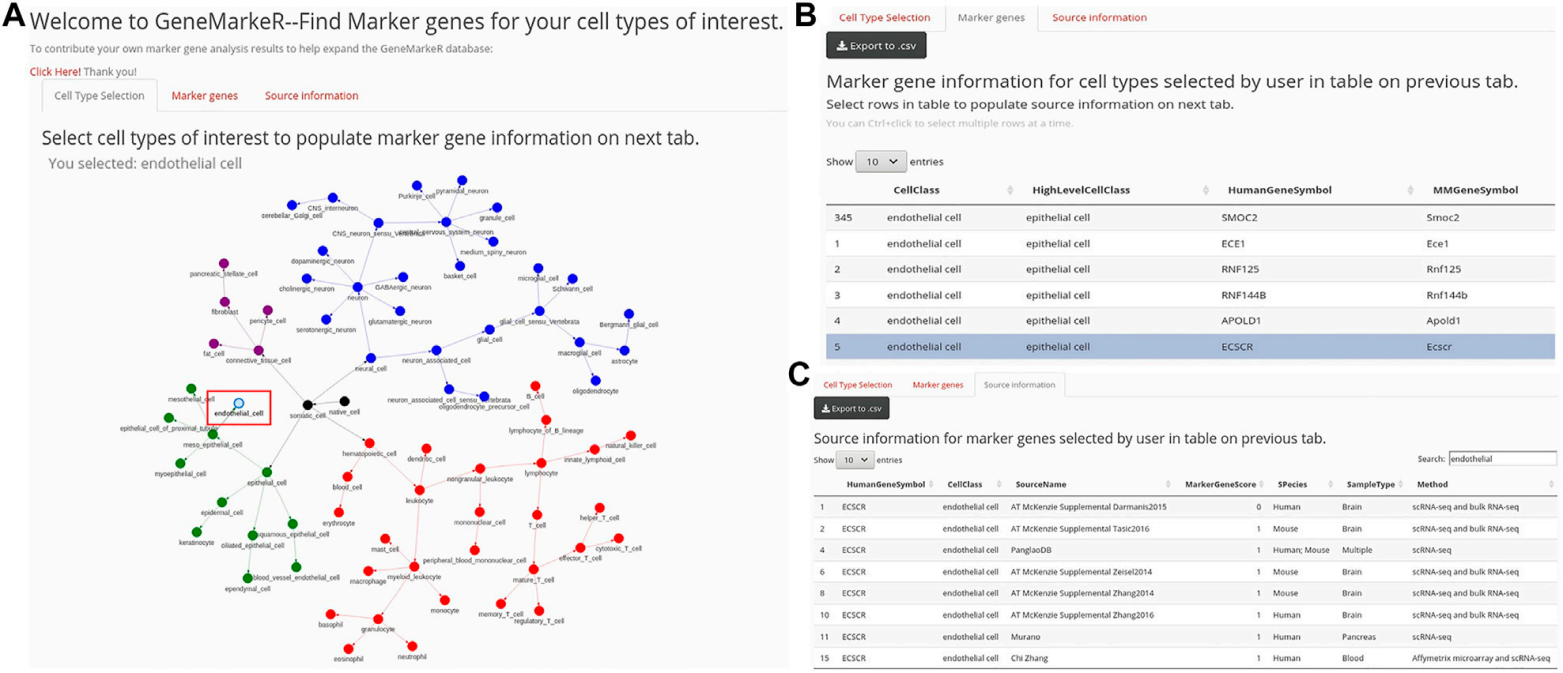
GeneMarkeR user interface. **(A)** “Cell Type Selection” tab enables selection of cell types from a drop-down menu (not shown) and a hierarchical cell network (abbreviated network shown here). Upon selection of cell types, the “Marker genes” tab is populated. **(B)** “Marker genes” tab displays marker genes and species specificity information from GeneMarker.db filtered to the user-selected cell types. Upon selection of marker genes of interest, the “Source information” tab is populated. **(C)** “Source information” tab displays source, sample and statistical data from CellSearcheR.db filtered to the user-selected marker genes. All GeneMarkeR data can be exported to CSV.

The 120 distinct cell types extracted from the publications were mapped to Cell Ontology terms using EMBL-EBI’s Ontology Lookup Service and Ontobee. Additional cell types were added to the network structure to ensure specific cell types accurately mapped back to parent nodes (i.e., naïve cell and somatic cell). Redundant terms (i.e., cell types that mapped in multiple branches) were pruned by removing cyclic relationships manually. Intermediate nodes that lacked branching and did not add value to the classification were manually removed. Intermediate nodes with branches were retained as these are crucial to build out the tree as cell types from new datasets are added. The cell type hierarchy of Cell Ontology was built via the JavaScript package “visNetwork” implemented in R with an abbreviated version shown in Figure 1A. The cell hierarchy enables us to consider if genes were specific for higher-level cell type terms vs. cell subtypes.

### Marker Gene Score

To compare disparate marker gene statistics across publications, each statistical endpoint from a source was normalized between 0 and 1. The midpoint (i.e., 0.5) was set as the author provided statistical significance cut-off. For example, in Supplementary Figure S1 the example Source 1 had two distinct statistical endpoints: 1) log fold change enrichment score, and 2) adjusted *p*-value. The log fold change enrichment score ranges from −9 to 0 where the more negative the result, the more significant. For log fold change enrichment score, these authors considered results less than or equal to −2 to be statistically significant; therefore, −2 is set at a marker gene score of 0.5 while values between −9 and −2 are scaled between 1 and 0.5, respectively and values between −2 and 0 are scaled between 0.5 and 0, respectively. The adjusted *p*-value for Source 1 ranged from 0 to 1, with increasing significance closer to 0. For adjusted *p*-value, these authors considered results less than or equal to 0.05 to be statistically significant; therefore, 0.05 is set at a marker gene score of 0.5 while values between 0 and 0.05 are scaled between 1 and 0.5, respectively and values between 0.05 and 1 are scaled between 0.5 and 0, respectively. The preliminary scores were averaged across the source per gene-cell type pair to calculate a marker gene score for each unique gene-cell type-source combination as is shown in Supplementary Figure S1. For example, in Supplementary Figure S1, if a unique gene-cell type pair are reported to have a log fold change enrichment score of −9 and an adjusted *p*-value of 0.05, then the preliminary scores of 1 and 0.5, respectively, would be averaged, resulting in a marker gene score of 0.75 for that gene-cell type pair in Source 1. A marker gene score of 1 indicates strong evidence that a gene is a marker gene for a given cell type from that source, while 0 indicates little to no evidence for supporting this relationship.

### Marker Gene Score Algorithm

**FIGURE 2:**
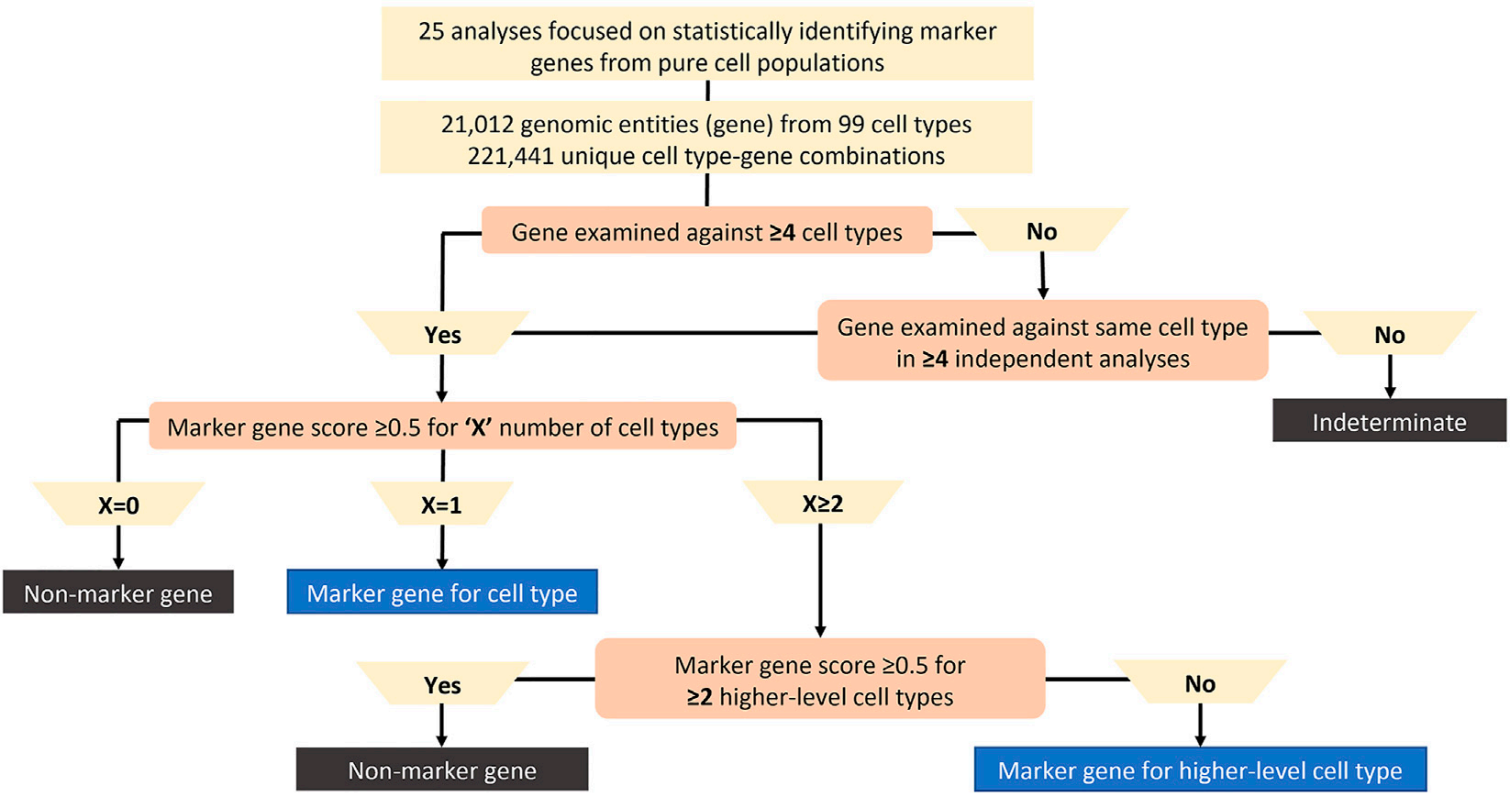
Marker gene classification algorithm. The algorithm classifies genes with <4 cell types as Indeterminate due to insufficient data to analyze the gene unless the gene was examined against the same cell type in ≥4 separate analyses. Significant marker gene score (i.e., at least 0.5) for at least two-thirds of publications considered for each gene-cell type pair. The number of significant gene-cell type pairs per a given gene is “X”. 1) If X = 0, the gene is not a marker gene for any cell type, 2) If X = 1, the gene is a marker gene for one cell type, 3) If X ≥ 2, then the gene is a marker gene for more than one cell type. When X ≥ 2, if the gene is marker gene for multiple cell types from the same higher-level cell type (ex: connective tissue cells), then the gene is a marker gene for the higher-level cell type. When X ≥ 2, if the gene is marker gene for multiple cell types from at least 2 higher-level cell types (ex: connective tissue cells and T cells), then the gene is a non-marker gene due to lack of specificity across publications.

To classify whether a gene was specific across samples, species and sources for a given cell type, a simple marker gene classification algorithm was developed as shown in Figure 2. Genes reported in fewer than 4 cell types were labelled as Indeterminate due to insufficient data to determine specificity across multiple cell types. As highly specific genes may not be expressed in other cell types accounting for reporting in fewer than 4 cell types, a subset of genes categorized as Indeterminate had to be reclassified. Therefore, genes originally classified as Indeterminate that were analysed in the same cell type across at least 4 separate sources for common cell types or 2 separate sources for rare cell types (e.g., pancreatic epsilon cell) were reclassified and were included in the next classification steps.

Next, the number of cell types (X) with an average marker gene score of ≥0.5 across publications were counted for each gene. If X = 0 for an individual gene, that gene was not considered a marker gene for any cell type (i.e., a non-marker gene). If X = 1 for an individual gene, that gene was significant for a single cell type across sources, so it was classified as a marker gene for that cell type. To ensure genes were specific for a limited number of cell types, each gene was restricted to be considered a marker gene for a maximum of 2 cell types. To ensure this cut-off was achieved, if X ≥ 2 for an individual gene, the number of higher-level cell types (Y) were considered. If Y < 2 for an individual gene, then the gene was a marker gene for the higher-level cell type. If Y ≥ 2 for an individual gene, the gene would be considered in most cases as a non-marker gene since it was not specific across publications. As each gene was restricted to a maximum of 2 cell types for which it was specific, genes exceeding this are labelled non-marker genes.

Therefore, using our algorithm cut-off X, we first check if the gene is specific for the more granular cell subtypes. If X < 2, then the gene is subtype specific, thus specific for that cell subtype and for any higher-level cell types in the hierarchical tree branch. While we count this gene as specific for 1 cell type subtype, the specificity relationship is propagated up the branch meaning the gene is also specific for higher level cell types in that branch. If X ≥ 2, then we check if those cell subtypes fall under the same higher-level cell type by looking at the built hierarchical ontology tree structure. This is where the higher-level threshold of Y comes into play. If a gene is found to be specific for multiple cell subtypes (i.e., X ≥ 2) and those cell subtypes belong to the same higher level cell subtype, then the gene is a marker gene for the higher-level cell type, but NOT for the subtypes. Species specificity for a marker gene required a 3-fold difference in median marker gene score between species with the median exceeding 0.5 for at least one of the species.

### Database Design and Web Interface

**FIGURE 3:**
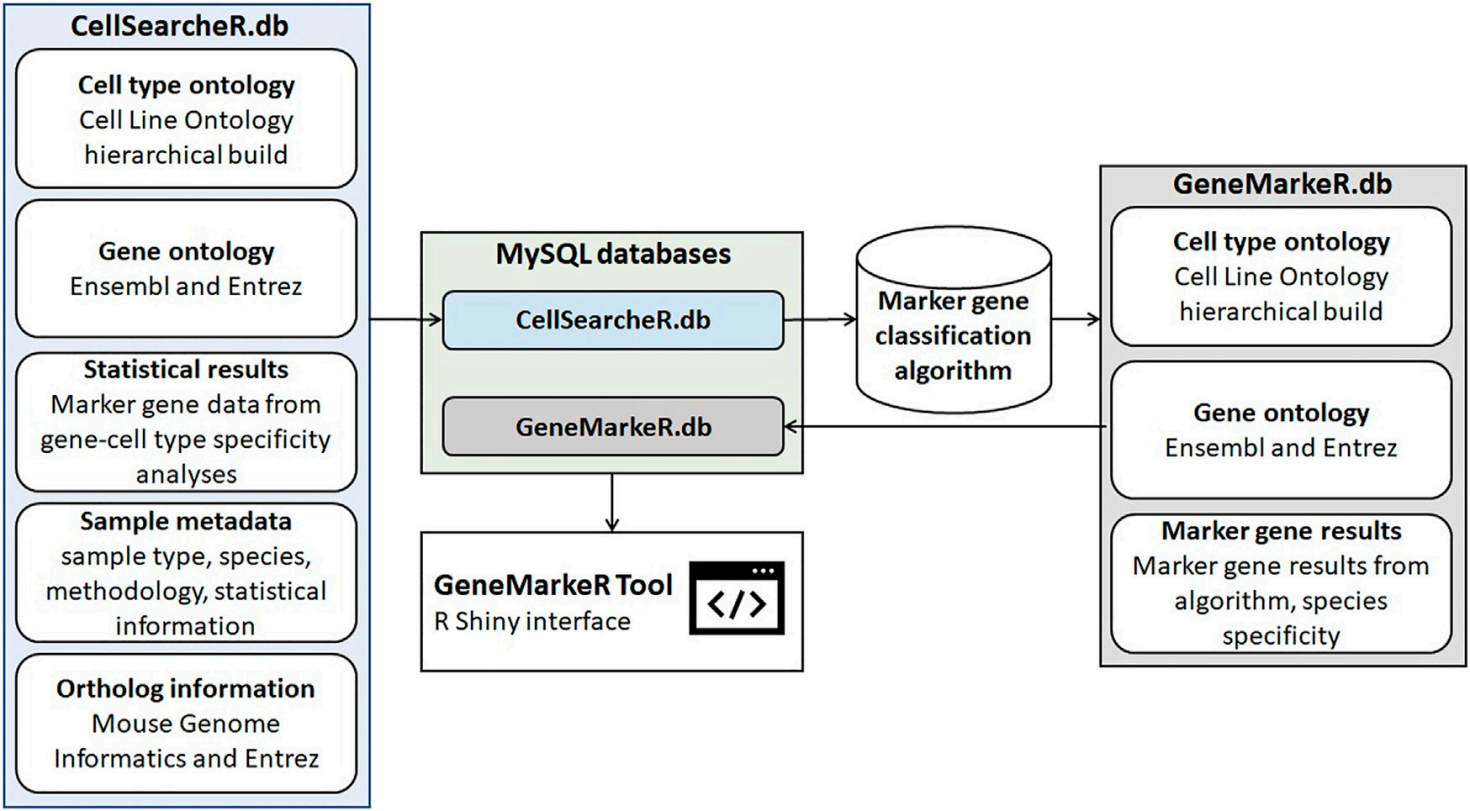
Schema for generation of GeneMarkeR. The CellSearcheR database (CellSearcheR.db) integrates standardized cell type, gene and statistical data from publications performing marker gene identification analyses on non-treated, non-disease mouse and human samples. Data was manually extracted, cell types mapped to the Cell Line Ontology hierarchical ontology, genes mapped to Ensembl and Entrez identifiers and statistical data normalized to a marker gene score. CellSearcheR.db is processed by the marker gene classification algorithm detailed in Figure 2. The GeneMarkeR database (GeneMarkeR.db) integrates the standardized cell type, gene and marker gene results from the algorithm output. CellSearcheR.db and GeneMarkeR.db are MySQL databases that are accessed via an R Shiny user interface called GeneMarkeR.

There are two databases behind GeneMarkeR shown in Figure 3, they are both implemented in MySQL to ensure data integrity, standardization, and ease of data updates over time. CellSearcheR.db consists of over 261,000 rows of data extracted across 15 publications and 2 datasets from collaborators comprising a total of 25 unique marker gene analyses. CellSearcheR.db was processed through the algorithm described in *Materials and Methods Marker gene score* to create GeneMarkeR.db, which stores gene-cell type relationships for the algorithm identified marker genes.

An R Shiny tool hosted on the IU Precision Health Initiative server enables access and extraction of both CellSearcheR.db and GeneMarkeR.db databases. As is shown in Figures 1A–C, the R Shiny tool has reactive programming built-in, so when the user selects cell types, this accesses GeneMarkeR.db to populate the marker gene tab with algorithm-derived marker genes for their cell types of interest. User selection of genes of interest on the marker gene tab reactively retrieves the standardized, raw CellSearcheR.db marker gene score and statistical data for each of those genes.

A link (https://redcap.uits.iu.edu/surveys/?s=XEAFCX4LC7) is provided on the web interface to a user submission form where researchers can submit their marker gene analysis data. The online form provides the results in a standardized CSV output to enable easy standardization and addition to CellSearcheR.db. In addition, marker gene analyses from new publications can also be manually extracted and standardized to update CellSearcheR.db with new data. The marker gene score algorithm is then used to process all the data in CellSearcheR.db to update the results in GeneMarkeR.db. Therefore, the process ensures updatability of the databases and web interface over time from user submission and manual extraction from new publications.

## Results

In total, 25 unique marker gene analyses of 9 distinct specimen types (blood, bone marrow, brain, heart, kidney, lung, pancreas, and tonsil) and additional cross-specimen sample types were identified that met the criteria specified in the *Materials and Method* section. The 261,000 rows of standardized marker gene statistical data extracted from the 25 analyses were stored in the CellSearcheR.db. As is shown in Figure 3, the CellSeatcheR.db data are analyzed in the marker gene classification algorithm detailed in Figure 2 to identify the marker genes that are then stored in GeneMarkeR.db. The information housed in each database is shown in Figure 3 and the data from both MySQL databases are used to generate the GeneMarkeR Tool R Shiny interface.

**FIGURE 4:**
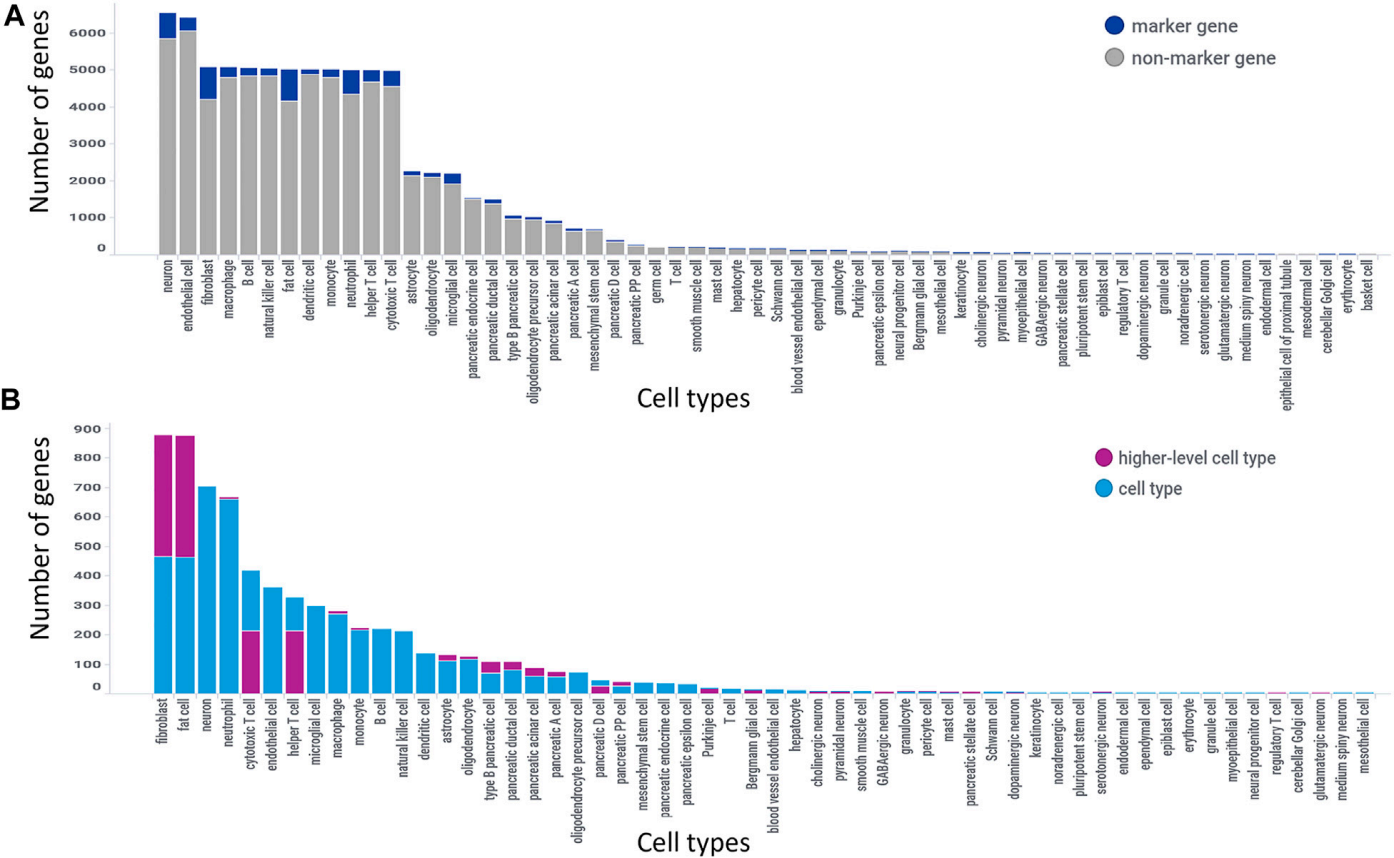
GeneMarkeR summary statistics. **(A)** Number of marker genes identified (dark blue) out of all genes analyzed per cell type (length of bar). **(B)** Number of marker genes reclassified as a higher-level cell type marker (purple) and number of marker genes classified as cell type-specific marker (light blue) out of all marker genes identified for that cell type (length of bar). Figure 2B is filtered to the marker genes, i.e., dark blue bars in figure 2A. Both figures are sorted from largest to smallest number of genes.

The 3,936 genomic entities that could not be automatically or manually mapped to a current gene annotation were excluded, leaving 21,012 genomic entities for the analysis. There were over 120 distinct cell types (including higher level cell types) with 221,441 unique gene-cell type combinations considered in the marker gene analysis. The final analysis of standardized marker gene results identified 2,464 genes as specific for one or two cell types with 2,746 total marker gene pairs as 281 genes were specific for two cell types. 7,283 genes were classified as non-marker genes, 10,465 were classified as indeterminate due to sparse data and the remainder were a mix of non-marker gene and indeterminate. The number of genes identified as a marker gene analyzed at that cell type (dark blue) out of all genes analyzed at that cell type (length of bar) is shown in Figure 4A. There were 68 cell types with marker genes identified out of the 120 cell types extracted from the 25 unique marker gene analyses. Out of the 2,746 marker genes, 80% of those were classified as a specific cell type and 20% were classified as a higher-level cell type. Filtering to the marker genes from Figure 4A (dark blue) we get Figure 4B where marker genes are categorized based on whether the gene is specific for that cell type (light blue) or a higher-level cell type (purple). For example, there were 5,000 genes analyzed against fibroblasts with approximately 500 being identified as marker genes for fibroblasts and 400 being identified as marker genes for a higher-level cell type (i.e., connective tissue cell).

In CellMarker there are an average of 2.2 sources supporting marker genes in normal tissue samples with 55% of marker genes supported by a single source. In the GeneMarkeR.db database there are 4.5 sources on average supporting a gene being a marker gene for a certain cell type in our database with only 4 (0.1%) marker genes supported by a single source. These 4 cases were due to the gene being a higher-level marker gene in the cell ontology and the individual publications having at least 4 distinct cell types to support that re-classification.

## Discussion

The analysis described here focused on mouse and human as these two species comprise most marker gene data analyses. Non-treated and non-disease samples were evaluated to study the naïve state of cell identity. This enables future analyses to delve into the impact that disease and treatment may have on cell identity markers. After extracting data from public datasets meeting these criteria, data standardization was addressed. Due to differences in genome annotations, sources of gene symbols, and naming conventions across publications, not all genes could be automatically mapped. Therefore, 15% of the gene symbols were manually mapped to current genome assembly GRCm39 for mouse and GRCh38.p13 for human. Genes that existed in earlier genome annotations but have since been discontinued in current mouse and human reference genomes were removed from the analysis.

The ontology standardization of cell type started with mapping cell types from the publications to Cell Ontology. Nodes of these cell types and their higher-level cell types were connected by building the network backwards from the most specific cell types up to the highest-level parent nodes (i.e., naïve cell or somatic cell). As is described in *Materials and Methods Ontology standardization*, the hierarchy was manually pruned to remove redundancy and circular relationships, while maintaining intermediate cell type nodes to ensure new cell types could be connected in the future. In a handful of cases nodes were manually adjusted to ensure biological relevance and consistency. The higher-level cell types were then added to the database to improve the marker gene score algorithm.

Due to differences in statistical methods, endpoints and significance cut-offs, the marker gene score was calculated to enable normalization and comparison across publications. Using the median and average marker gene score we used the marker gene score algorithm to identify marker gene, higher-level marker gene, non-marker gene and indeterminate calls for each cell type-gene combination across sources. While approximately 80% of gene-cell type pairs could be automatically annotated by following the algorithm, genes with more than 2 higher-level cell types had to be manually checked to determine if those higher-level cell types were from the same branch of the hierarchical cell map or from a previously pruned branch. For example, microglial cell can be connected to multiple branches (i.e., glial cell, macrophage, and myeloid cell, etc…), so the manual mapping would reconsider these additional connections and higher-level cell types in context of all data for that gene-cell type pair.

While CellMarker is a great source of marker gene annotations from normal and disease samples, the database described in this manuscript provides an improvement in marker gene identification for normal mouse and human samples. An advantage of this algorithm over previously published analyses is the greater amount of data supporting each marker gene call. Identifying genes that are considered as marker genes across multiple sources in CellMarker requires users to perform their own analysis of the data, whereas GeneMarkeR provides the user with that information. In addition, unlike CellMarker, GeneMarkeR considers the difference and overlap between mouse and human enabling species-specific gene markers to be included or excluded. Finally, due to the inclusion of hierarchical cell ontology in GeneMarkeR, 538 genes were more accurately reclassified as being specific for a higher-level cell type rather than the original publication cell type, which is not considered in CellMarker.

In conclusion, data were first manually extracted from publicly available marker gene analyses and hierarchical ontology standardization was applied to create CellSearcheR.db. Next, GeneMarkeR.db was developed using a novel algorithm that considers marker gene score to identify marker genes specific across species, samples, and methodology. Finally, an R Shiny user interface was developed (GeneMarkeR) that pulls from CellSearcheR.db and GeneMarkeR.db using reactive programming. The GeneMarkeR tool provides highly validated, consistent marker genes and species specificity information to enable improved scRNA-seq cell type identification over existing databases.

## Data Availability

The original contributions presented in the study are included in the article/Supplementary Material, further inquiries can be directed to the corresponding author. GeneMarkeR is freely available at https://shiny.ph.iu.edu/GeneMarkeR/ access on the web with all major browsers supported.

## Supplementary Material

Supplementary Figure 1 Marker gene score calculation for an individual source/publication. For each marker gene statistic in an individual source, the statistical results are scaled from 0 (low/no evidence marker gene) to 1 (high evidence marker gene) using the author specified statistical significance cutoffs. All gene-cell type pairs in that source receive a preliminary score for each endpoint. Those preliminary scores are then averaged to calculate the marker gene score for a gene-cell type pair in an individual source. A given gene-cell type pair will have a different marker gene score for each publication to enable comparison of gene specificity for that cell type across sources.
