# Supplementary figures and images for: GeneMarkeR: A Database and User Interface for scRNA-seq Marker Genes
Source: Front Genet. 2021 Oct 26;12:763431. doi: 10.3389/fgene.2021.763431 (PMC8577352; doi:10.3389/fgene.2021.763431)

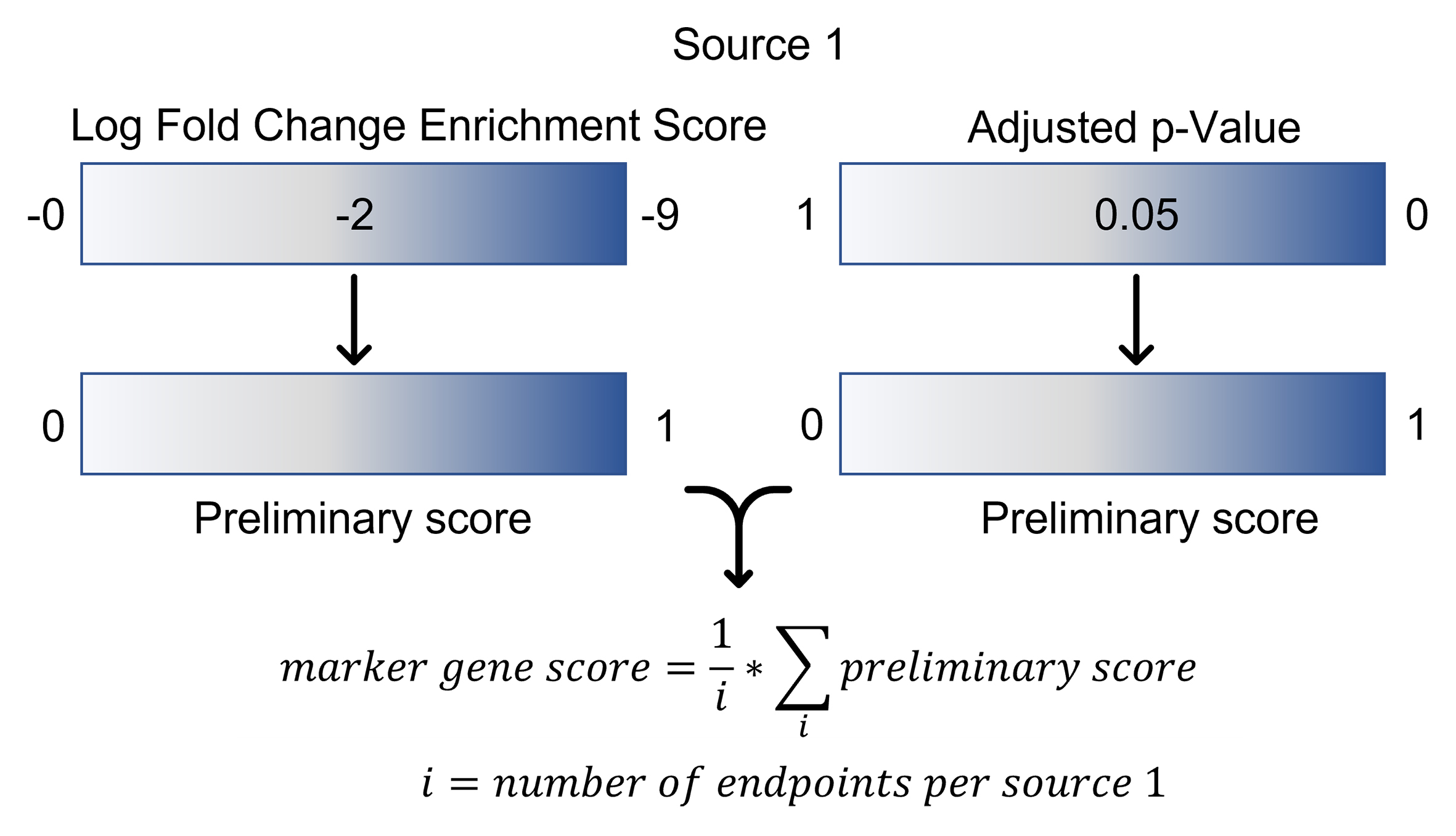

Supplement: Supplementary file 1 [file Image1.JPEG]
